# Clinical Features and Course of Patients with Peripheral Exudative Hemorrhagic Chorioretinopathy

**DOI:** 10.4274/tjo.71354

**Published:** 2016-10-17

**Authors:** Zafer Cebeci, Yasemin Dere, Şerife Bayraktar, Samuray Tuncer, Nur Kır

**Affiliations:** 1 İstanbul University İstanbul Faculty of Medicine, Department of Ophthalmology, İstanbul, Turkey

**Keywords:** Hemorrhage, retinal pigment epithelial detachment, age-related macular degeneration

## Abstract

**Objectives:**

To evaluate the clinical characteristics of patients who were followed in our clinic with the diagnosis of peripheral exudative hemorrhagic chorioretinopathy (PEHC).

**Materials and Methods:**

Medical records of 12 patients who were diagnosed with PEHC in İstanbul University İstanbul Faculty of Medicine, Department of Ophthalmology between July 2006 and June 2014 were reviewed retrospectively.

**Results:**

This study included 21 eyes of 12 patients. Four (33.3%) of the patients were male and 8 (66.7%) were female and ages ranged between 73 and 89 years. Eight (66.7%) of the patients were referred to us with the diagnosis of choroidal mass. Unilateral involvement was found in 3 and bilateral involvement in 9 patients. Temporal quadrants were involved in all eyes. Fifteen eyes (71.4%) had subretinal hemorrhage and hemorrhagic/serous retinal pigment epithelial detachment, 11 (52.4%) had lipid exudation, 5 (23.8%) had chronic retinal pigment epithelium alterations, 2 (9.5%) had subretinal fibrosis and 1 (4.8%) had vitreous hemorrhage. PEHC lesions were accompanied by drusen in 11 eyes (52.4%), geographic atrophy in 2 eyes (9.5%), and choroidal neovascularization scar in 2 eyes (9.5%). Treatment was done in both eyes of a patient for lesions which threatened the macula, in a patient with bilateral macular edema and in a patient with vitreous hemorrhage. The remaining eyes were followed-up without any treatment because the lesions did not threaten the macula and they showed no progression during follow-up.

**Conclusion:**

PEHC is a degenerative disease of peripheral retina that is seen in older patients, and signs of age-related macular degeneration (AMD) may accompany this pathology. Especially in patients with AMD findings, the peripheral retina must be evaluated carefully for existing PEHC lesions.

## INTRODUCTION

Peripheral exudative hemorrhagic chorioretinopathy (PEHC) is a disease of the peripheral retina that emerges with advancing age. PEHC is characterized by exudation and hemorrhages and can often be mistaken for an intraocular mass.^1^ In 1962, Reese and Jones^2^ first reported patients who exhibited sub-retinal pigment epithelium (RPE) hematoma in the peripheral fundus and accompanying age-related macular degeneration (AMD). Silva and Brockhurst^3^ described patients with a similar clinical picture and called the disease ‘peripheral RPE hemorrhagic detachment’. In 1980, Annesley^4^ named the condition of subRPE and/or subretinal hemorrhage with subretinal exudation as the term used today, ‘PEHC’. PEHC is more common in older patients who are female and white.^5,6^ Though the etiology of this pathology remains unclear, its defining features are peripheral subretinal and/or subRPE hemorrhage, exudation and retinal pigment epithelial detachment (PED), and the lesions are often located in the temporal quadrant.^5,6^

The disease is usually static or spontaneously regresses, leaving a fibrotic scar. However, lesions that progress from the periphery to the macula result in declines in visual acuity. Signs of AMD such as drusen, RPE alterations and choroidal neovascularization (CNV) may also accompany the disease.^5^

The aim of this study was to evaluate the clinical characteristics, follow-up and treatment outcomes of patients diagnosed with PEHC in our clinic.

## MATERIALS AND METHODS

The medical records of patients diagnosed with PEHC between July 2006 and June 2014 in the İstanbul University İstanbul Faculty of Medicine, Department of Ophthalmology were analyzed retrospectively. PEHC diagnosis was based on the presence of subRPE and/or subretinal exudation or hemorrhage and/or the appearance of a mass in the extramacular peripheral retina. Patients with history of ocular trauma, intraocular inflammation or tumor and those with other systemic or ocular diseases which may cause retinal hemorrhage or exudation were not included in the study.

Data regarding age, gender, duration of symptoms, systemic diseases and medications used were recorded for all patients. Initial and final best corrected visual acuity (BCVA), anterior segment and fundus examinations, and intraocular pressure were evaluated. Patients with pronounced appearance of a mass were examined by ultrasonography (USG). Fundus photographs were taken. The presence of active PEHC lesions in both eyes was considered symmetric bilateral disease, while the presence of active disease in one eye and findings of RPE atrophy and sequelae in the peripheral retina of the fellow eye was considered asymmetric bilateral disease. Fundus fluorescein angiography (FFA) and indocyanine green angiography (ICGA) were done in selected patients. The macula was evaluated by spectral domain optical coherence tomography (OCT).

The study was designed and conducted in accordance with the principles of the Declaration of Helsinki.

Data were analyzed by paired samples t-test using Statistics Package for the Social Sciences (SPSS) version 20.0 (IBM Corp., Armonk, NY, USA) software. P values less than 0.05 were accepted as statistically significant.

## RESULTS

The patients’ demographic and clinical characteristics are presented in Table 1. Of the 12 patients (21 eyes) included in the study, 4 (33.3%) were men and 8 (66.7%) were women. Mean age was 82.4 (range, 73-89) years. Mean follow-up time was 15.6 (range, 6-74) months. Eight patients (66.7%) were referred to our clinic with suspected choroidal mass. Mean duration of symptoms was 2.8 months (range, 1 week-12 months). The most common complaint at presentation (in 7 patients, 58.3%) was reduced vision. Four patients (33.3%) reported having metamorphopsia and 2 patients (16.7%) reported pain. Ten patients (83.3%) had systemic hypertension and 5 (41.7%) had diabetes mellitus. Eight patients (66.7%) had a history of using oral anticoagulants.

The right eye was involved in 1 patient, the left in 2 patients, and the remaining 9 patients had bilateral involvement. Of those with bilateral involvement, 4 (33.3%) had symmetric bilateral disease and 5 (45.7%) had asymmetric bilateral disease. Mean BCVA was 0.81±0.92 (0.00-2.6) logMAR at presentation and 0.73±0.84 (0.00-2.6) logMAR at final examination. The difference between initial and final BCVA was not statistically significant (p=0.39).

**Figure 1:**
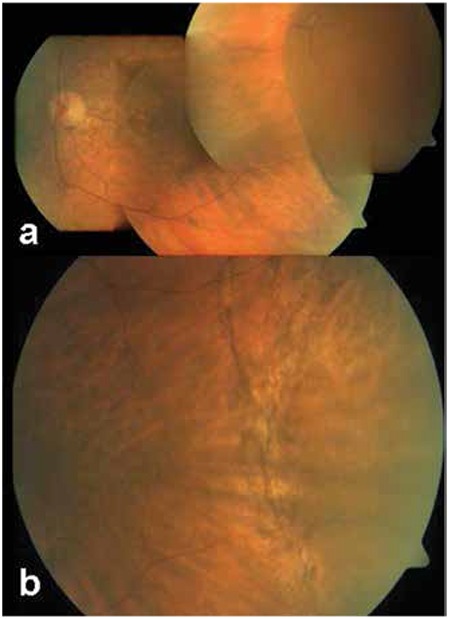
Images from an 87-year-old female patient referred for a choroidal mass in the left eye, a) macular drusen and serous retinal pigment epithelial detachment in the temporal periphery, b) appearance after spontaneous regression of pigment epithelial detachment during follow-up without treatment

**Figure 2:**
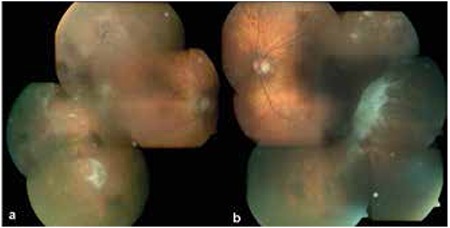
Color fundus photography of the eyes of an 89-year-old male patient, a) subretinal hemorrhage, exudation and subretinal fibrosis are visible in the temporal and inferior periphery of the right eye, b) subretinal fibrosis and hemorrhage are visible inferotemporally and exudation is apparent superotemporally in the left eye

Lesions were observed in the temporal quadrant in all cases, and 2 patients (16.6%) also exhibited involvement in the nasal quadrant. Lesion involvement extended from the peripheral retina anteriorly to the midperipheral retina. Subretinal hemorrhage and/or hemorrhagic/serous PED was detected in 15 eyes (71.4%), lipid exudation in 11 eyes (52.4%), chronic RPE alterations in 5 eyes (23.8%), subretinal fibrosis in 2 eyes (9.5%) and intravitreal hemorrhage in 1 eye (4.8%) (Figures 1 and 2). The appearance of a mass was detected USG in 4 (19%) of the patients referred for further evaluation of a suspected choroidal mass.

Macular involvement was observed in 14 eyes (66.7%). Macular lesions were detected by ophthalmoscopic examination and OCT. Fifteen eyes (52.4%) had drusen, 3 (14.3%) had macular edema, 2 (9.5%) had geographic atrophy, 2 (9.5%) had CNV scar and 1 (4.8%) had epiretinal membrane. A patient with drusen in both eyes (patient #3) also exhibited macular edema in both eyes at presentation. This patient was treated for exudation extending to the macula in the left eye. In other eyes with drusen and geographic atrophy, the peripheral retinal hemorrhage or exudation had not reached the macula.

A patient whose vision level was hand motions and had intravitreal hemorrhage at presentation was treated with a single intravitreal bevacizumab (IVB) injection (1.25 mg/0.05 mL). The intravitreal hemorrhage resolved and the patient’s vision improved to 20/20. A patient with bilateral involvement whose peripheral lesions threatened the macula in both eyes was treated with 3 IVB (1.25 mg/0.05 mL) injections in the right eye and 1 IVB injection in the left eye and with 2 sessions of photodynamic therapy (6 mg/m^2^ verteporfin-50 J/cm^2^) applied to midperipheral lesions in the left eye. A bilateral PEHC patient with macula edema in both eyes was treated with intravitreal 0.5 mg/0.1 mL ranibizumab in 3 injections to the right eye and 7 injections to the left eye. Treatment was not recommended for another patient with macular edema because the patient’s vision level was counting fingers at 50 cm. Sixteen eyes (76.2%) were followed without treatment because the peripheral lesions did not threaten the macula or there were no active lesions.

**Figure 3:**
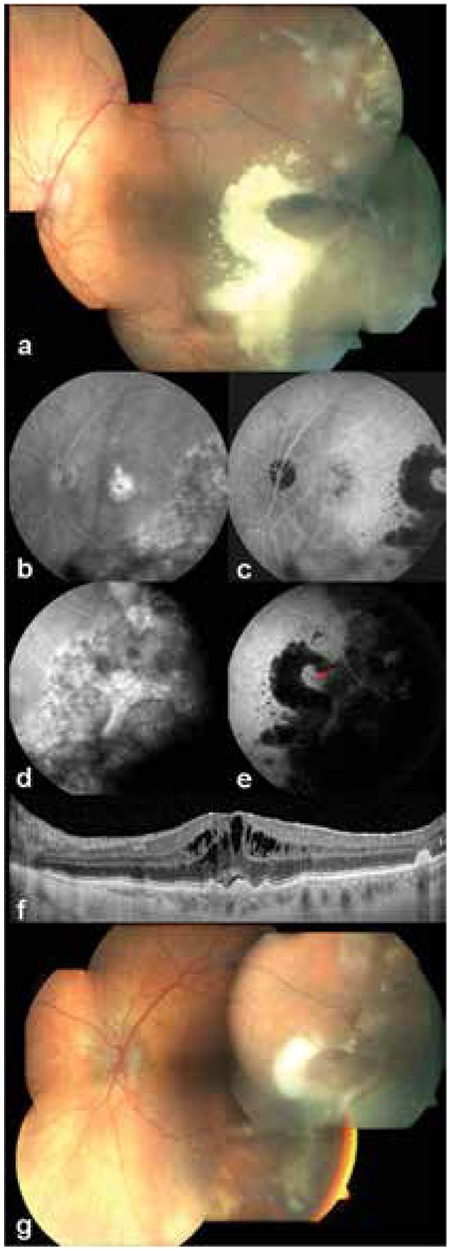
Images from an 86-year-old female patient who presented with reduced vision in the left eye, a) extensive exudation and subretinal hemorrhage in the temporal periphery, b) macular edema and hyperfluorescence due to temporal leakage are evident on fundus fluorescein angiography, c) temporal area of extensive exudation shows hypofluorescence on indocyanine green angiography, d) the temporal area shows hyperfluorescence on fundus fluorescein angiography, e) appearance of polyps (red arrow) in the center of the temporal hypofluorescent area on indocyanine green angiography, f) drusen and subretinal and intraretinal fluid are apparent on optical coherence tomography, g) exudation and hemorrhages partially regressed after seven intravitreal ranibizumab injections

Four patients underwent FFA and 3 patients underwent both FFA and ICGA. Areas of serous PED showed diffuse hyperfluorescence which became more pronounced in the late phase, while areas consistent with hemorrhages showed hypofluorescence in both phases. ICGA revealed polypoid lesions in the temporal quadrant in one patient, who was treated due to macular involvement (Figure 3).

## DISCUSSION

PEHC is a rare degenerative disease of the peripheral retina predominantly seen in elderly women and accompanied by hemorrhage and/or exudation.^4^ In a study by Mantel et al.^5^ including 45 patients, 68.9% were women and their ages ranged from 60 to 91. Another series of 143 PEHC patients determined that 67% were women and the average age was 80.6 Consistent with the literature, 66.7% of the patients in our study were women whose mean age was 82.4 years. Women have a longer life expectancy compared to men and thus they more often survive to the advanced age at which the disease becomes symptomatic, explaining the predominance of women in this pathology.^5^

Higher rates of systemic hypertension and systemic anticoagulant have been observed in PEHC patients.^1,6^ We found that 83.3% of our patients had systemic hypertension and 66.7% used an anticoagulant agent. Fluctuations in systemic blood pressure and long-term use of anticoagulation are believed to be risk factors for hemorrhage, one of the main features of PEHC, and are suspected to have a role in recurrent hemorrhage.

PEHC lesions are usually located peripherally between the equator and the ora serrata in the temporal quadrant and anterior retina.^1,5,6^ Nasal lesions often extend to the temporal quadrant as well, or may be present as separate lesions accompanying temporal lesions.^5^ All of our patients exhibited temporal involvement of the peripheral retina. However, there is no evidence-based explanation for the predominance of lesions in the temporal quadrant.

Reduced visual acuity commonly occurs when peripheral subretinal hemorrhage, fluid or lipid exudation advances to the macula, or in the presence of intravitreal hemorrhage.^5^ In addition to macular involvement of peripheral lesions, vision level may also be affected by macular edema and accompanying AMD-related findings such as drusen, CNV and geographic atrophy. Shields et al.^6^ observed age-related macular changes in 17% of same eyes and 27% of contralateral eyes in PEHC patients. In a 2009 study, Mantel et al.^5^ detected AMD in 31 of 56 eyes and macular edema in 4 eyes, and later reported in a 2012 study that 12 of 48 eyes had AMD findings and 6 had macular edema.^1^ In the present study, we observed accompanying AMD findings in 15 of 21 eyes. One of the main features of PEHC is onset at advanced ages, which may explain the frequency of accompanying signs of AMD and the additional macular findings in these patients. Furthermore, checking the peripheral retina is often neglected during macular examination of AMD patients, leading to potential PEHC lesions being overlooked. Thorough peripheral examinations for additional pathologies should definitely be performed in AMD patients at every examination.

The natural course of the disease is generally stable, or spontaneous regression may occur with atrophy, fibrosis or hyperplasia.^6^ Shields et al.^6^ observed spontaneous regression in 89% of 173 eyes. Of the 76.2% of eyes in the current study that did not have active macular involvement and were followed without treatment, none showed lesions in new areas or clinical advancement or progression of existing lesions.

PEHC treatment options reported in the literature include monitoring, photocoagulation, photodynamic therapy, cryotherapy, intravitreal anti-vascular endothelial growth factor (anti-VEGF) injection and vitrectomy.^7,8^ Following without treatment is recommended for cases without affected central vision or macular involvement, and the disease may spontaneously regress in most of these cases.^9^ Laser photocoagulation or photodynamic therapy may be applied in cases with macula-threatening findings or peripheral polyps.^10^ Though rare, the development of vitreous hemorrhage or massive subretinal hemorrhage may require pars plana vitrectomy.^9^ Alforja et al.^7^ treated an eye with PEHC-related subretinal neovascularization and subfoveal fluid with a single IVB injection (1.25 mg/0.05 mL) and observed that the subfoveal fluid and lesion resolved, leaving subretinal fibrosis. In a larger study, Pınarcı et al.^8^ evaluated 23 eyes with PEHC of 15 patients and reported that lesions were stable or regressed in 11 patients (47.8%). They treated 12 patients with IVB injection, and 9 eyes (39.1%) showed lesion regression with atrophy and scarring after 2 or 3 injections. Visual acuity decreased in the other 3 patients due to macular involvement despite repeated injections.^8^ The efficacy of intravitreal ranibizumab injection against PEHC-associated lesions has also been demonstrated.^11^ We observed no reduction in vision during follow-up in our patients without macular involvement or macula-threatening lesions that we monitored without treatment. There was no significant change between initial and final visual acuities in our study. This may be due to the lack of PEHC-related macular involvement or CNV-like active macular pathology in patients who were followed without treatment and to the preservation of initial vision level in patients who underwent treatment. However, it is not possible to reach a definitive judgment regarding functional outcomes due to the small patient group and the low proportion of that group that underwent treatment.

In addition to diagnosing PEHC based on patient history and ophthalmoscopy, FFA and ICGA can assist in diagnosis. FFA can reveal hypofluorescence consistent with hemorrhage in lesion areas, and irregular hyperfluorescence shown by CNV and homogenous hyperfluorescence typical of serous PED in the periphery. In their 2009 study in which 20 eyes were examined by ICGA, Mantel et al.^5^ did not find polypoidal choroidal vasculopathy (PCV) in any of the eyes but detected pathologic choroidal vascular networks in 6 eyes. However, in their 2012 study using ultra-wide angle ICGA in 48 eyes, they found polypoid formations in 69% of the eyes and abnormal choroidal vasculature in 50%.^1^ As in PCV, choroidal vascular disorders may also play a role in PEHC. Like the serous or hemorrhagic PED and subretinal hemorrhage or exudation that may be seen in both pathologies, the presence of recurrent hemorrhage is another common factor that suggests a similar mechanism between the two conditions.^12^ Therefore, it has been proposed that PCV and PEHC are actually two aspects of the same disease, or that PEHC may be a unique variation of type 1 neovascularization that results from PCV.^9,12^

Differential diagnosis of PEHC should include peripheral chorioretinal lesions such as retinal capillary hemangioma, retinal macroaneurysm, retinal telangiectasia, choroidal hemangioma, choroidal melanoma and choroidal detachment.^13^ In a study evaluating 12,000 patients, PEHC was identified in 13% of patients referred with a diagnosis of uveal melanoma, and all 143 PEHC patients had been referred with an initial diagnosis of choroidal melanoma.^6,14^ In Mantel et al.’s^5^ series, 86.6% of 56 eyes presented with an initial diagnosis of choroidal malignancy. In the present study, 66.7% of our patients were referred to our clinic with an initial diagnosis of choroidal mass. Important features of PEHC that allow its differentiation from melanoma, the most common misdiagnosis, are retinal exudation, presence of diffuse macular and peripheral RPE alterations, absence of sentinel vessels on anterior segment examination, appearance of hypofluorescence on FFA, and absence of intrinsic vascular pulsation on USG.^6^

Limitations of our study include the small study population, retrospective analysis, and lack of FFA and ICGA data for all patients.

PEHC is a rare age-related disease of unknown etiology which can lead to reduced vision in patients with subfoveal exudation and/or hemorrhage accompanied by age-related degenerative changes. These patients should be examined regularly for sight-threatening macular pathologies, and examinations should include a careful evaluation of the peripheral retina as well as the macula in patients with AMD findings. Studies with larger patient groups may elucidate the etiology of PEHC and facilitate the selection of appropriate treatment methods.

### Ethics

Ethics Committee Approval: This study retrospective, Informed Consent: It was taken.

Peer-review: Externally peer-reviewed.

## Figures and Tables

**Table 1:**
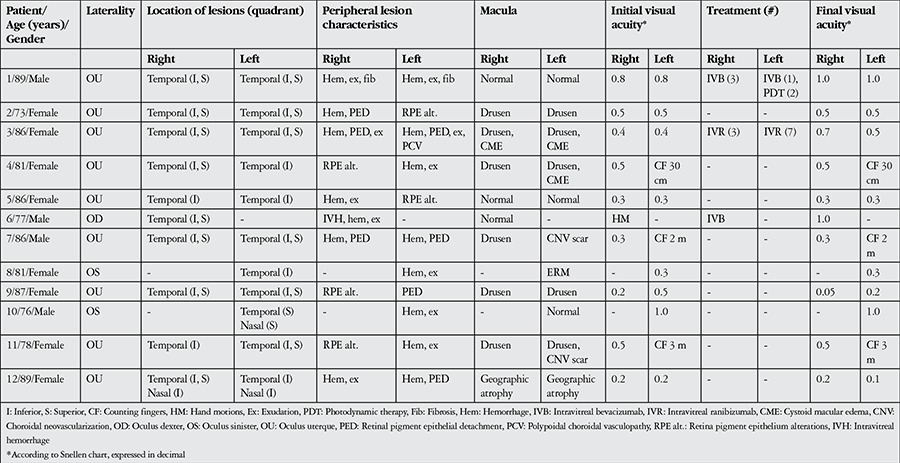
Characteristics of patients with peripheral exudative hemorrhagic chorioretinopathy
